# Rationale for Environmental Hygiene towards global protection of fetuses and young children from adverse lifestyle factors

**DOI:** 10.1186/s12940-018-0385-y

**Published:** 2018-04-23

**Authors:** Jean-Pierre Bourguignon, Anne-Simone Parent, Jos C. S. Kleinjans, Tim S. Nawrot, Greet Schoeters, Nicolas Van Larebeke

**Affiliations:** 1 Pediatric Endocrinology, CHU Liège, 600, rue de Gaillarmont, B-4032 Chênée, Belgium; 2 Neuroendocrinology Unit, GIGA Neurosciences, University of Liège, Quartier Hôpital, Tour 4 - 1er étage, 15 Avenue Hippocrate, B-4000 Liège, Belgium; 3 Department of Toxicogenomics, Maastricht University, Maastricht, The Netherlands; 4 Centre for Environmental Sciences, Hasselt University, Hasselt, Belgium; 5 Centre for Environment and Health, Leuven University, Leuven, Belgium; 6 Flemish Institute for Technological Research (VITO), Mol, Belgium; 7 Department of Biomedical Sciences, University of Antwerp, Antwerp, Belgium; 8 Department of Environmental Medicine, Institute of Public Health, University of Southern Denmark, Odense, Denmark; 9 Department of Radiotherapy and Experimental Cancerology, Ghent University, Ghent, Belgium; 10 Department of Analytical, Environmental and Geo-Chemistry, Vrije Universiteit Brussel, Brussels, Belgium

**Keywords:** Pregnancy, Mutagens, Endocrine disrupting chemicals, Carcinogens, Precautionary principle, Public health, Developmental origin of health and disease

## Abstract

**Background:**

The regulatory management of chemicals and toxicants in the EU addresses hundreds of different chemicals and health hazards individually, one by one. An issue is that, so far, the possible interactions among chemicals or hazards are not considered as such. Another issue is the anticipated delay of several decades before effective protection of public health by regulatory decisions due to a time consuming process. Prenatal and early postnatal life is highly vulnerable to environmental health hazards with lifelong consequences, and a priority period for reduction of exposure. There are some initiatives regarding recommendations for pregnant women aiming at protection against one or another category of health hazard, however not validated by intervention studies.

**Hypothesis:**

Here, we aim at strengthening the management of exposure to individual health hazards during pregnancy and lactation, with protective measures in a global strategy of Environmental Hygiene. We hypothesize that such a strategy could reduce both the individual effects of harmful agents in complex mixtures and the possible interactions among them. A panel of experts should develop and endorse implementable measures towards a protective behavior. Their application is meant to be preferably as a package of measures in order to maximize protection and minimize interactions in causing adverse effects. Testing our hypothesis requires biomonitoring studies and longitudinal evaluation of health endpoints in the offspring. Favorable effects would legitimate further action towards equal opportunity access to improved environmental health.

**Conclusion:**

Environmental Hygiene is proposed as a global strategy aiming at effective protection of pregnant women, unborn children and infants against lifelong consequences of exposure to combinations of adverse lifestyle factors.

**Electronic supplementary material:**

The online version of this article (10.1186/s12940-018-0385-y) contains supplementary material, which is available to authorized users.

## Background

### Prenatal/neonatal exposures and lifelong consequences

For about four decades, the human population has been exposed to an increasingly large array of synthetic chemicals. Only about 1% of those chemicals have been studied so far since scientific research is time-consuming and costly [1]. They include mutagens, Endocrine Disrupting Chemicals (EDCs), carcinogens and teratogens that may cause life-long harm depending on life period and level of exposure among other factors [2]. Past findings and derived concepts indicate that several adult diseases represent late onset consequences of early exposures [3–6]. A pioneering dramatic illustration was the occurrence of vaginal cancer and reproductive disorders in the offspring of mothers treated with diethylstilbestrol during pregnancy [3, 7]. Here, transgenerational and other studies point toward involvement of epigenetic mechanisms [8]. Another pioneering observation was the possible fetal origin of testicular cancer [9, 10]. This provided the basis of the Testicular Dysgenesis Syndrome linking delay in differentiation of fetal testes with lifelong consequences including reduced sperm quality and testicular cancer [4]. Early exposures to EDCs can have huge impact on development and on the risk of diseases such as adult reproductive failure, cancer, obesity, diabetes and metabolic syndrome, and neurodevelopmental disorders among others [11]. Fetal exposure to dietary carcinogens seems to induce molecular events that indicate increased cancer risks together with other adverse health effects such as reduced birth weight and head circumference [5]. Childhood cancer, in particular leukemia among boys, can be causally related to the maternal dietary intake of carcinogenic substances during pregnancy [5]. Fetal exposure to mutagens such as polycyclic aromatic hydrocarbons also increases the risk of cancer and neurodevelopmental disorders [12]. Telomeres, markers of biological ageing are highly variable at birth and it has been identified recently that maternal exposures to air pollution is associated with telomere length of the next generation [13]. Taken together, those data demonstrate some causal mechanisms linking early life exposures and later health. Besides these examples of early disorganization of health for the rest of life, fetal life is also a critical period due to occurrence of unique processes such as brain development. As an example, disruption of thyroid hormone promotion of brain development during fetal and early postnatal life has detrimental consequences on lifelong intellectual abilities [14]. Overall, a robust set of data concurs to support prioritization of pregnancy and early postnatal life for a healthy environment [15, 16]. All those findings are consistent with the concept of Developmental Origin of Health and Disease (DOHaD) [17]. This concept was promoted by the observation that impaired fetal growth, a reflection of intra-uterine exposure to adverse conditions in the maternal environment, can be predictive of adult metabolic malfunctioning [18, 19]. However, behind the different observations discussed here along the DOHaD concept, different mechanisms can possibly be involved and deserve studies in each specific condition.

### Regulatory management of hazardous chemicals in the European Union

**Fig. 1:**
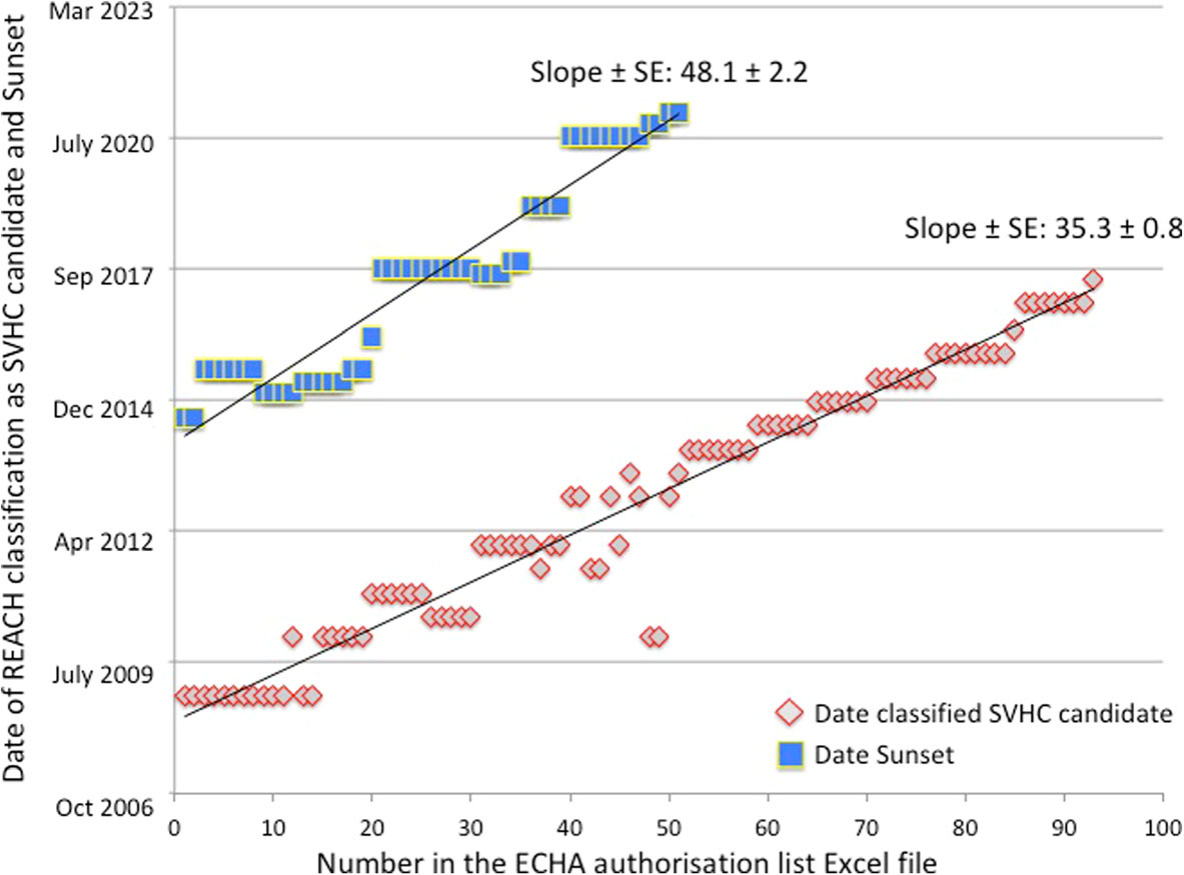
For each substance identified in the authorization list under REACH regulation, two dates are represented: when the substance was identified as of very high concern (SVHC) candidate and the date of sunset i.e. when the substance has been or will be phased out following the regulatory decision. The regression lines and the slopes ± Standard Error are shown. The slopes are significantly different (F test, *p* < 0.0001). The data were retrieved from https://echa.europa.eu/authorisation-list and https://echa.europa.eu/candidate-list-table (accessed 15 September, 2017)

The development of a regulatory framework for the management of chemical substances in the European Union (EU) has been rightly viewed as a progress, hopefully contributing to reduced exposures including in early life. For example, REACH in 2006 [20] and the more recent regulations for plant protection products in 2009 [21] and biocidal products in 2012 [22] have provided the tools for chemical’s risk management. While the health risk is a function of exposure, the first step in a strategy of limiting exposure is the identification of the hazard. The REACH regulation, which applies since 2008, allows action under its authorization regime: a hazardous substance can be included in the candidate list, i.e. identified as of very high concern (SVHC) and subsequently included in the so-called “authorization” list, i.e. banned as of a sunset date [23]. The data on these two regulatory actions [24, 25] indicate that there is on average a 7-year time span between the moment a substance has been identified as a SVHC and the moment it is being phased out. This time span however appears to increase with time (Fig. 1) as indicated by the slopes of the regression lines which are significantly different (F test, *p* < 0.0001). The time span is longer (F test, *p* < 0.001) for substances identified as SVHCs in the period 2011–2013 (7.67 ± 1.41 yrs., mean ± SD) than 2008–2010 (6.46 ± 0.69 yrs). Between October 2008 and June 2013, 52 substances have been regulated as SVHC accounting for 10 chemicals regulated each year. The regulatory decision about those 52 chemicals refers most frequently to carcinogenicity (*n* = 28) and toxicity for reproduction (*n* = 14), not excluding associated endocrine disrupting properties such as observed with phthalates [11]. Also shown in Fig. 1, there are 42 substances that have been identified as SVHCs between December 2013 and July 2017 [25] but no decision to phase them out has been taken so far [24]. Time since registration was not considered in this analysis since the date of registration was biased by differences in both the criteria for registration and time since marketing the substance.

The EU laws for identification and regulation of chemicals have set a new scene for long debates between stakeholders including industry, public authorities, non-governmental organizations (NGOs) and scientists, among others. A recent illustration is provided by the scientific criteria for identification of EDCs [26, 27]. While the current paradigm of management of individual hazardous factors is a requirement and must be pursued, it is a very slow process. So far, 1409 chemicals (last updated September 2017) have been listed as potential EDCs based on data published in the peer-reviewed literature [28]. Since this estimate does not include carcinogens and mutagens, we hypothesize a likely underestimated figure of 1–2% hazardous chemicals among the 145,297 chemicals listed by ECHA as pre-registered before 2008 (last updated 11 August 2017). Based on the observed regulation of 10 chemicals per year under REACH and assuming a similar figure for the chemicals not falling under REACH, several generations would likely be needed before the possible carcinogens, mutagens, repro-toxic and EDCs are effectively regulated.

## Presentation of the hypothesis

During the first half of the twentieth century, the implementation of a global anti-microbial hygiene led to an important decrease in the morbidity and mortality of infectious diseases, before the identification of most pathogenic microbial agents and the advent of antibiotics [29]. An analogous strategy, Environmental Hygiene, a physical-chemical hygiene aiming at limitation of exposure to hazardous agents, in particular mutagenic agents and EDCs, is proposed here to reduce the burden of those factors present in environment. We hypothesize that, during prenatal and early postnatal life as a priority period for intervention, a global protective approach (Environmental Hygiene) could effectively reduce some complex exposures. Consequently, adverse health effects resulting from action of individual agents as well as interactions among them could also be reduced. It is hoped that such a global strategy will save time and protect health while awaiting that a healthy environment becomes a reality through the regulatory measures. The suggested approach is consistent with the precautionary principle and should involve regulatory authorities and industry in information of the public and the professionals towards equal opportunity access to improved environmental health.

**Fig. 2:**
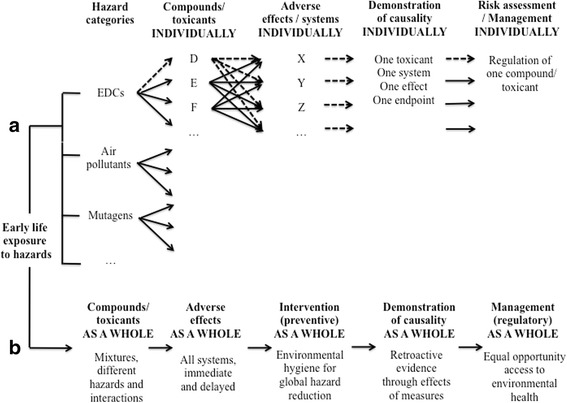
Two complementary paradigms for the management of factors hazardous to human health are illustrated. The current paradigm (panel **a**) and the proposed additional paradigm (panel **b**) are schematically illustrated. Along the current strategy, the dashed arrows indicate that, based on a single hazardous factor (D), different systems and adverse effects are considered (X, Y, Z, …), each deserving demonstration of causality before risk is assessed and the hazardous factor managed. The approach of the issue as a whole is meant to reduce interactions among hazardous factors, save time before hazard reduction and contribute to equal opportunity access to environmental health

In Fig. 2, the sequence of events is schematically illustrated and compared in the current regulatory approach of individual health hazards (Fig. 2, panel a) and in the proposed strategy of Environmental Hygiene (Fig. 2, panel b). As shown in panel a (Fig. 2), regulation identifies different categories of health hazards e.g. mutagens, EDCs, carcinogens and teratogens. In each category, compounds or toxicants (D, E, F…) are considered individually through their effects on a given system (X, Y, Z, …) e.g. reproductive, thyroid/neurodevelopmental, metabolic/obesogenic, as recommended by OECD [30]. A compound or toxicant can affect different systems through involvement of different endpoints in each system. The critical demonstration of causality is provided by the study of one effect caused by one toxicant on one endpoint in one system, individually. When sufficient evidence has accumulated, risk assessment and management of each particular compound or toxicant are performed. Along the strategy of Environmental Hygiene (Fig. 2, panel b), the hazardous factors, the adverse effects, the intervention and the causality are addressed globally. Considering exposure to health hazards as a global condition is consistent with the exposure to environmentally relevant mixture of chemicals and the resulting interaction between chemicals and categories of hazards e.g. chemicals and psychosocial stress. Evaluation of the adverse effects as a whole can integrate immediate and delayed effects in different systems together. Here, the demonstration of causality is not a prerequisite to a preventive intervention as a whole. The concept is development of Environmental Hygiene for global reduction of exposure to hazards. It is suggested that an international panel of experts should develop and endorse relevant and implementable protective measures. Their application is intended to be preferably as a package of measures in order to maximize protection from exposures and to minimize interactions among hazards in causing adverse effects. The demonstration of causality is meant to be a global and retroactive process. Intervention studies are warranted with biomonitoring and longitudinal evaluation of health endpoints in the offspring. Based on the evidence obtained, the issue of equal opportunity access to improved environmental health will have to be addressed by authorities to make health protection available to all pregnant women and unborn children through action such as training of health professionals and consumer information.

## Implications of the hypothesis

### Number of hazardous compounds and factors

The raising *number of compounds* to be evaluated in each category of hazardous factors vastly out-pace scientific studies about those compounds [1]. Despite efforts towards development of high throughput tests for mutagenicity and interaction of individual chemicals with different endocrine axes (e.g., reproduction, thyroid, energy balance), data about many chemicals are completely missing. Also, an approach “chemical by chemical” is not consistent with the environmentally relevant exposure to low-dose mixtures that account for complex effects [31, 32]. Incorporation of those findings in the decision-making process is challenging since the management of chemicals is meant to be one by one. An emerging issue is also that different factors with different modes of action can synergize and interact in causing adverse effects [32]. An example is tumor promotion, abundantly studied through in vivo experiments [33] and possibly responsible for the human cancer risk after multiple exposure involving dioxins and dioxin-like substances [34–36]. The concern of exposure to combination of chemicals raises several issues. At the very beginning of life, synthetic chemicals from different classes can be quantified already in cord blood and in samples from pregnant women or of reproductive age [37–39]. Overall, the fetus can be exposed to more than 300 chemicals. As analytical techniques improve, it is expected that many more environmental chemicals will be identified in human fluids and tissues. It is not known how these chemicals interact and at what exposure levels these combinations may pose health risks. Risk assessment of combined exposures is on the agenda of the European Commission who asked the European Food Safety Authority (EFSA) to develop a strategy for assessing health risks related to combined exposures [40]. One strategy is to group chemicals that belong to the same chemical class such as PCB congeners or dioxins. Chemicals can be grouped because they act on the same target and form a cumulative assessment group as proposed for pesticides by EFSA [41]. Alternatively exposures may be concurrent when chemicals are present in the same products. A more holistic approach is that specific lifestyle, behaviors and environmental settings may also lead to high exposures to a number of pollutants and high risks in vulnerable groups such as the unborn, children or socio economic deprived subpopulations. Possible combinations of adverse lifestyle factors involve non-chemical hazards. For instance, exposure to a stressful event during pregnancy can have cumulative effects with chemicals [42, 43]. Thus, studies addressing each factor one by one will often underestimate both hazard and risk, signaling the requirement of more studies evaluating the effects of different factors together.

The proposed global strategy addresses *different compounds or factors as a whole*. This approach is likely to involve various hazardous chemicals or factors identified in the environment (air, drinking water) and in consumer products (e.g. food, drinks, home care and personal care). Work environment should also be taken into account. Identification of hazardous factors in relation with products and environmental conditions aims at building simple recommendations that probably reduce exposure. This approach will address the issue of low-dose mixtures and combination of different health hazards since application of several protective measures as a package will likely reduce the mechanistic interaction among the agents or hazards. Chemical hygiene may be efficient to reduce multiple exposures in vulnerable groups.

### Evaluation of adverse effects

The classical *evaluation of adverse effects* (as recommended by OECD) considers the different systems separately i.e. male hormones (androgens), female hormone (estrogens), thyroid hormones, hormones controlling weight and glucose metabolism, etc. [30]. However, many hazardous chemicals lack specificity of interaction and can affect different parts of the endocrine system [11]. The neuroendocrine effects of Bisphenol A provide an illustration of the complexity and non-specificity of adverse effects [44]. Importantly, the action of a given hazardous factor on a given hormone in vivo results in reactive changes in the same hormonal system or axis (e.g. feedback mechanisms) and cross-talking between different axes, e.g. leptin and reproduction [45, 46]. Such mechanistic components can be missed when addressing adverse effects using components of the endocrine system one by one.

Along the proposed strategy, the *adverse effects will be addressed as a whole*. This kind of approach is including together different endpoints or outcomes that belong to different systems. This multisystem approach emancipates scientists and regulators from linking a single chemical exposure to a single adverse outcome, and is consistent with the reality of involvement of different systems in the in vivo conditions of exposure to mixtures of hazardous factors. This includes the interaction between hazardous factors in causing some effects as well as the interaction between systems in explaining an effect or a reaction to an effect.

### Preventive intervention against hazardous factors

The central and original component in the proposed global approach is *preventive intervention against hazardous factors as a whole* that is not subordinate to thorough demonstration of causal involvement of each individual factor in adverse effects. Environmental Hygiene aims at global reduction of exposure to hazards, especially in pregnancy and early postnatal life. Implementation of Environmental Hygiene should start as early as possible in pregnancy. Starting before pregnancy would have been a preferable option because pre-pregnancy health weighs significantly on pregnancy outcomes and clearance of persisting pollutants. While such an extension is worth being implemented in the future, we have considered that the pregnant status is associated with increased likelihood of changing consumer behaviors in an initial phase and that focusing on pregnant women would improve feasability. Recommendations aiming at pregnancy have been published by Governmental agencies e.g. the Danish Environment Protection Agency [47] or non-governmental organizations. We suggest that an international panel of experts should develop and endorse the protective measures. The panel should be multidisciplinary including gynecology, pediatrics, endocrinology, toxicology, public health and epidemiology among others. Environmental Hygiene is meant to provide guidelines validated by experts based on our current knowledge of effects of individual hazardous factors. Preliminary studies will have to show that they are implementable. Examples of such measures are provided in Table 1. Specific comments and references to each recommendation can be found in the Additional file 1.

**Table 1:** Some recommendations aiming at reduced exposure to health hazards during pregnancy and early postnatal life

	Recommendations	Targeted hazards		
		EDCs	Mutagens	Others
Everywhere	Stop smoking tobacco and drinking alcohol	x	x	x
	Limit as much as possible passive smoking	x	x	
	Avoid frequent close presence to power lines; limit the use of cell phones or cordless mobile phones			x
	Limit the use of plastic or rubber toys and prefer products declared to be free of bisphenol A or phthalates	x		
	Stay in a cool place in case of heat > 30 °C			x
Personal care	Restrict the use of cosmetics and lotions as much as possible	x		
	Prioritize unscented products and stop using perfumes	x		
	Do not color your hair; do not polish your nails	x		
	Avoid tattoos		x	
Food and drinks	Prioritize food and drinks from glass container instead of plastic bottles or metal cans	x		
	Do not microwave food in plastic recipients	x		
	Use quality-controlled water in glass bottles	x		x
	Prioritize organic food whenever possible	x	x	
	Avoid processed, especially nitrite treated, meat		x	
	Avoid charred meat and consumption of bread or other cereal products that are darkened due to high temperature treatment		x	
	Limit (once a week) consumption of predator fish (tuna, swordfish, …)	x		x
Home care	Wash new clothes before wearing them	x	x	x
	Avoid exposure to organic solvents		x	
	Avoid as much as possible painting or coating (walls, doors, floors, …)	x	x	
	Avoid scented cleaning products, air fresheners and fragrances	x	x	x
	Clean inside the house using damp clothes and reduce dust	x		
	Do not use insecticides	x		
	Ventilate the bedrooms and living rooms at home for 10 min, 1–2 times a day	x		x
Outdoor	Avoid the use of herbicides or pesticides	x	x	
	Close the car windows and recycle air while driving on highways, in tunnels and in heavy traffic		x	x
	Prefer exercising in green areas and avoid heavily polluted air such as within 200 m of heavy traffic		x	x
Others	Avoid exposure to medical x-rays unless really necessary		x	x

### Demonstration of causality

In the regulatory management of chemicals one by one, science is expected to provide the *demonstration of causal involvement* of a given chemical before any measure is considered. Carrying the burden of proof is challenging since most human health disorders that are possibly involving adverse effects of chemicals are multifactorial [11]. This, together with the exposure to chemicals as mixtures, explains why only a limited fraction of a given effect can be attributed to a given chemical. Attribution of a given effect to a mixture and elucidation of the respective contribution of agents in the mixture effect is even more challenging given the number of compounds and the variety of mechanisms. Moreover, for ubiquitous compounds, there is no unexposed population that can provide an estimate of the “baseline” prevalence of disease to which chemicals may contribute an additional burden. Human epidemiology plays a critical role but carries severe limitations due to exposure to mixtures, possibly long latency to effects, variability in unintended level of exposure and negative confounding due to exposure of the control population to other factors having the same effects, among other reasons. While the generally agreed-upon WHO definition of EDC [2, 11] states that the adverse effect is a consequence of altered function of the endocrine system following exposure to the chemical (or mixture), the EU Commission has introduced in the scientific criteria a focus on the endocrine mode of action of which the adverse effect is a consequence [48]. These requirements undoubtedly will add to the delay in decision – making. Diethylstilbestrol and PCBs were banned several decades ago while our understanding of their mode of action was minimal as compared to nowadays.

Along our proposed strategy, the *demonstration of causality is meant to be a retroactive process.* Namely, the proof of the causal role of the hazardous factors is not a prerequisite to the global reduction of exposure. Instead, demonstration of the favorable impact of the global protective measures on the level of mother and offspring exposure studied by biomonitoring together with the effects on a number of health indicators will provide evidence of global causality. An intervention is substantiated by the numerous studies on the causal link between a given factor and a given adverse effect. Intervention studies are rather scarce such as a recent study on the effect of dietary recommendations on exposure of pregnant women to methyl mercury in Denmark [49]. While available studies on causal involvement of individual hazardous chemicals legitimate the global approach, development of more intervention studies is desirable though limited by ethical reasons and other factors such as possible latency of decades between exposure and effects. The mode of action does not appear to be a prerequisite in the global approach. Also, the intervention does not aim at a given product from a given company and intervention is not contingent upon demonstration of causal involvement of a given chemical. However, the possible demonstration of favorable effects on health outcomes after reduced exposure to some hazards through Environmental Hygiene will challenge industry to demonstrate that chemicals that they produce are not involved.

### Risk assessment and management

A final step in the classical management of hazardous chemicals is *risk assessment.* Here, the dose is meant to be critical in an attempt to define a so-called safe dose. This approach is raising several issues including the possible gaps between in vitro models and in vivo conditions, variations in sensitivity to chemicals depending on endpoints and life periods as well as possible non-monotonic dose-response relationship [26, 50]. All those factors complicate the evaluation of risk and account for additional time needed before regulatory decision.

### Involvement of stakeholders towards the pregnant woman as ultimate actor

**Fig. 3:**
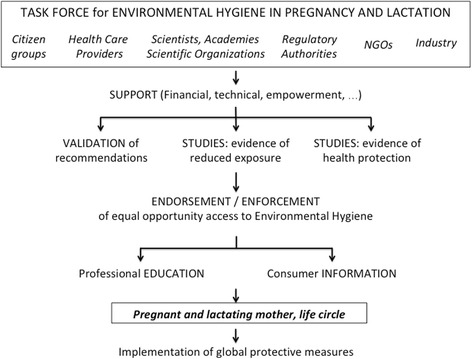
Implementation of Environmental Hygiene. A task force involving the different stakeholders is proposed and provides support to the initiative including financial, technical and any other aspects. The task force clarifies the role of stakeholders in subsequent action including validation of recommendations aiming at protection of pregnant and lactating mothers from environmental hazards and setting up studies aiming at evidence of reduced exposure and health protection in the offspring. Based on those studies, the task force endorses and enforces the strategy of Environmental Hygiene that must be made available to all. The next steps are professional education of health care providers and consumer information, with pregnant women and their life circle as ultimate actor

The perspective and the implementation of Environmental Hygiene could unduly pressurize pregnant women. A mother should not blame herself for poor outcomes that must be attributed to collective negligence of industry, policymakers and others. Conversely, safer outcomes should result from mobilization of many stakeholders providing support and action towards women in pregnancy as the ultimate actors. A strategy is proposed in Fig. 3. We suggest that a *task force* binds together the different stakeholders in developing support to the initiative. This includes financial and technical means as well as empowerment of the different stakeholders in the different actions required for implementation of Environmental Hygiene. The next step consists of testing the hypothesis through validation of the recommendations and studies aiming at evidence that Environmental Hygiene can reduce exposure and protect health. These issues are addressed in the next section. The proposed strategy will then lead to *action towards equal opportunity access to improved environmental health*. The article 2 of the Treaty on European Union [51] states « The Union is founded on the values of respect for human dignity, freedom, democracy, equality, the rule of law and respect for human rights, including the rights of persons belonging to minorities. These values are common to the Member States in a society in which pluralism, non-discrimination, tolerance, justice, solidarity and equality between women and men prevail ». This substantiates action towards equal opportunity access to improved environmental health. Provided that scientific studies validate the benefits of a global approach, policymakers will have to ensure that access to Environmental Hygiene is not limited by educational, socio-economic or any other characteristic of subpopulations [52]. For instance, proper *information of consumers* about the composition of products will be critical. This is a regulatory issue implying that labeling is consistent with composition, readable and understandable. Moreover, *education of health care providers*, particularly those taking care of pregnant women and young children, should be developed in terms of both content and information tools [53]. The key of that management proposal is the individual citizen i.e. the individual pregnant woman and young parents who deliberately become players for the protection of their offspring and possibly next generations.

Environmental Hygiene will be conducted in conjunction with the current *management of individual hazardous chemicals* by regulatory authorities. This process aims at banning or restricting the use of a given chemical. The resulting benefits can take decades due to data gaps required to prove causation, time consuming experimental or epidemiological work, debates between stakeholders and persistence of some chemicals in the environment among other reasons. The regulatory evaluation of chemicals remains however a keystone in the management of hazards and risks threatening public health. It is therefore critical that policymakers take any suitable measures that can speed up the process of chemical safety assessment and management. The regulatory process is beyond the control of individual citizens and health care providers and may dismiss preventive management, a feeling reinforced by discordant information about the impact of chemicals on human health, and insufficient education. Industry also has a crucial role in the quality of raw materials used in the preparation of consumer products. This is essential for the presence or absence of hazardous chemicals [5]. This issue is beyond the awareness of consumers including pregnant women and advisers such as health care professionals. Awareness requires transparent and readable information about constituents in consumer products. Therefore, industry has a very important initial role that must be implemented and monitored by authorities.

The issues of Environmental Hygiene far transcend Europe. They have been addressed globally by WHO in a recent publication [54]. WHO points to emerging environmental hazards including chemicals as a threat to children’s health and proposes a precautionary approach to protecting children from the effects of chemicals. This important work is symbiotic with our hypothesis and legitimates extension of the efforts to a global scale.

## Testing the hypothesis and concluding remarks

Environmental Hygiene is proposed as a global strategy aiming at protection of pregnant women, unborn children and infants against hazardous factors as a whole. Three research questions can be identified about the proposed strategy and must be addressed by scientists with financial support from public authorities: 1. What could be consensual and implementable protective measures in pregnancy and lactation? 2. What is the evidence that those protective measures reduce exposure to hazardous chemicals? 3. What is the evidence that those protective measures improve health? Implementation of such studies will have to address several issues including selection of recommendations, monitoring of exposure to hazards and health outcomes. The panel of experts will have to identify the criteria used for selection of the relevant recommendations. These criteria should incorporate the likelihood of reduced exposure through the proposed measure as well as the applicability based on the psychosocial characteristics of the study population. Questionnaires and interviews will be crucial for assessment of consumer behaviours before and during the study. The parameters selected for biomonitoring of exposure before and during the study will depend on baseline consumer behaviours, access to biological material and reliability of measurements among other factors. Inevitably, the studied population will be heterogeneous as far as the baseline consumer behaviours and exposures. Information on the efficacy of individual protective measures can come out of well-designed observational studies in a population of pregnant women. They could be stratified for specific lifestyles that they plan before or in early pregnancy and that they effectively embrace during pregnancy. These data could be used for the purpose of comparison with an intervention study using Environmental Hygiene as a package of measures. Inclusion of a control group is likely not feasible because everyone is exposed to some hazards and for ethical reasons. Some questions arise from the likely differences in risk awareness and health impact among the consumer behaviors. For example, the very serious consequences of fetal exposure to mother smoking and drinking alcohol and the public awareness about those issues may justify that refraining from smoking and drinking alcohol is an inclusion criterion in all the study groups. The inclusion criteria should be selected to maximize the chance of demonstrating the effects on exposure and health outcomes. An example is a short term intervention study of exposure to BPA and phthalates where the selected subjects were those reporting the most frequent use of canned foods [55]. The recruitment of subjects is challenging as shown in a study on reduction of mercury exposure in pregnant women [56]. These authors were able to enrol 8% or 36% of the women contacted by mail or directly approached on the ward before a scan, respectively. The investigators will have to motivate the participants, for instance through the feedback on exposures before and after implementation of Environmental Hygiene. Over the past 10 years, birth cohorts embraced the wave of new omics technologies to allow and understand the molecular pathways from exposure towards disease prevention. Environmental Hygiene in early life will benefit from omics as a tool to address causality along with the aforementioned classical concepts, even on the basis of observations. Based on the results of such studies, all stakeholders could endorse Environmental Hygiene and the strategy should become accessible to all. Such an objective will need joint action of academies, regulatory authorities and NGOs towards education of health care providers and consumer information.

The production of many environmental hazards arises out of economic activity, and the consequences of Environmental Hygiene cannot be ignored. While government inaction is often justified out of a concern that regulatory measures can stunt economic growth, the economic benefits are likely to be great, given the substantial disease burden that can be prevented by reducing exposure. Endocrine disruptor-related diseases are well known to contribute costs on the order of 1.2 and 2.3% of Gross Domestic Products in Europe and the US, respectively [57]. Among these costs, mixtures of EDCs were identified as contributors to disease-related costs, and a global approach is likely to maximize the economic impacts. Relevant exposures are also known to cluster by routes and categories of exposure (e.g., food packaging, pesticides), and a single contaminant approach is less likely to maximize effects on hormonal pathways (e.g., thyroid) that are particularly important.

Environmental Hygiene can by no means substitute for regulatory management restricting or banning chemical use. Such a regulatory approach is indispensable to protect public health in the long term and to reduce detrimental effects of chemicals on animal and plant biodiversity. However, Environmental Hygiene calls for additional involvement of regulatory authorities in information and education of consumers and professionals towards global protective behaviors and equal opportunity access to improved environmental health.

## Additional file

Additional file 1: Comments and references to each of the recommendations aiming at reduced exposure to health hazards during pregnancy and early postnatal life. (DOCX 61 kb)

## Abbreviations

CDC: Center for Disease Control and prevention
DOHaD: Developmental Origin of Health and Disease
ECHA: European Chemicals Agency
EDC: Endocrine Disrupting Chemical
EFSA: European Food Safety Authority
EU: European Union
NGO: Non Governmental Organization
OECD: Organisation for Economic Co-operation and Development
REACH: Registration, Evaluation, Authorisation and Restriction of Chemicals
SD: Standard Deviation
SVHC: Substance of Very High Concern
UNEP: United Nations Environment Program
WHO: World Health Organization
