# Supplementary material for: Rationale for Environmental Hygiene towards global protection of fetuses and young children from adverse lifestyle factors
Source: Environ Health. 2018 Apr 23;17:42. doi: 10.1186/s12940-018-0385-y (PMC5914065; doi:10.1186/s12940-018-0385-y)
Supplement: Supplementary file 1 — Comments and references to each of the recommendations aiming at reduced exposure to health hazards during pregnancy and early postnatal life. (DOCX 61 kb) [file 12940_2018_385_MOESM1_ESM.docx]

**Rationale for environmental hygiene towards global protection**

**of fetuses and young children from adverse lifestyle factors**

Jean-Pierre Bourguignon^1,2^, Anne-Simone Parent^1,2^, Jos CS Kleinjans^3^, Tim S. Nawrot ^4,5^, Greet Schoeters^6,7,8^ and Nicolas Van Larebeke ^9,10^

ADDITIONAL FILES

Examples of measures towards global protection of fetuses and young children from adverse lifestyle factors are provided in Table 1B together with some references. They are not comprehensive and meant to provide some evidence linking exposures (prenatally whenever studied) with adverse health effects. Some recommendations are general and relevant everywhere. Smoking tobacco and passive smoking in pregnancy are a known risk factor for many diseases including central nervous system tumors and disordered child neurodevelopment [1-5]. Maternal alcohol consumption is also associated with disturbances of child and adolescent neurodevelopment [3, 4]. Close exposure to power lines appears to be associated with childhood leukemia [6, 7]. Maternal exposure to mobile phone frequency electromagnetic fields is associated with behavioral and speech problems in children [8, 9]. Many toys and baby products contain EDCs such as Bisphenol A and phthalates that leak and contaminate the users prominently during the first hours of use [10]. High ambient temperature can trigger adverse birth outcomes, such as preterm birth and low birth weight [11].

Some recommendations aim at personal care. Chemicals with endocrine disrupting properties are added to cosmetics as preservatives (triclosan), fragrances (polycyclic musks), vehicles (diethyl phthalate), UV screens (benzophenone-3) or as a plasticiser in nail polish (dibutyl phthalate). Their metabolites can be measured in urine samples of a general population including children and the levels are associated with the reported use of personal care products [12, 13]. Synthetic fragrances are ubiquitous components of personal care and household cleaning products, they contain constituents such as phthalates, paraben, glutaraldehyde, hydroperoxides, oil of turpentine, metals, nitro musks, and essential oils, among others [14]. These compounds have also endocrine disrupting properties and are easily taken up by dermal contact as well as inhalation of contaminated dust and volatilized fragrances. In a French study reviewing 28 cosmetic products used by pregnant women, only half of them considered that cosmetics carry a possible risk and only 7-10% changed the use of nail polish or hair dye [15]. Tattooing might contribute to the risk of cancer as modern tattoos cause high skin concentrations of hazardous azo pigments, some being carcinogenic [16].

Some recommendations aim at food and drinks including exposure due to chemical leakage from plastic recipients and metal cans. There is some evidence that prenatal exposure to bisphenols and phthalates is followed by increased risk of behavioral and respiratory disorders in childhood [17, 18] as well as possible male reproductive and cardio-metabolic disorders in adulthood [19, 20]. The presence of agricultural chemicals and arsenic in drinking water is linked with increased occurrence of birth defects [21]. Prenatal exposure to persistent organic pollutants is known to be associated with adverse neurodevelopmental effects [22]. Consumption of organic food during pregnancy can result in a reduced risk of maternal obesity and diabetes [23] and decreased prevalence of hypospadias in the offspring [24]. Consumption of processed meat especially nitrite-treated, was reported to be associated with increased risk of cancer [25] (Bouvard et al. 2015) such as consumption of charred meat and darkened bread [26, 27]. (Fu et al. 2011; Kleinjans et al. 2015).

Home care also deserves some recommendations. In a recent study of the Swedish Chemical agency, 2,400 substances of health concern were linked to textiles [28] (Swedish Chemicals Agency 2016). More than 200 substances, as for example acid-type dyes, may contribute substantially to allergic skin reactions. Of major concern are azo dyes that are associated with increased risk of cancer and developmental effects. Reproductive toxicity has mainly been associated with flame retardants, phthalates, highly fluorinated water, stain repellants and biocide treated textiles. Laundering will release some of the chemicals depending on how they are bound to textiles [29, 30]. Some organic solvents are known to cause cancer and maternal exposure to solvents is associated with childhood leukaemia and lymphoma [31, 32]. A registry based case control study in Scandinavian countries (n cases/controls: 8112/26264) showed a small but significant increase in testicular germ cell tumors of sons when the mother was exposed to toluene one year prior to birth [33]. During pregnancy, parents often redecorate their homes. Especially changing floor materials, has been shown to increase exposure to hazardous chemicals and increases the risk for respiratory diseases in early childhood especially in families with a history of atopic diseases [34]. Air fresheners emit hazardous components such as benzene, phthalates and limonene and may form secondary pollutants due to reaction with ozone. Health risks include damage to the central nervous system and alteration of hormone levels [35]. The EU EPHECT project (Emissions, Exposure Patterns and Health Effects of Consumer Products in the EU) showed irritating and respiratory effects associated with consumer products used indoors. Combinations of purpose/kitchen/floor cleaning agents, furniture/floor polish, combustible/electric air fresheners, and perfume contributed considerably to formaldehyde emissions [36]. House dust carries chemicals such as phthalates, flame retardants, synthetic fragrances [37] and also lead, cadmium, pesticides, PAHs, bacteria, allergens. Babies are much more exposed than adults and much more sensitive to the health effects of dust making it important to reduce indoor dust by efficient cleaning practices [38]. Exposure to insecticide spraying during pregnancy was found to be associated with a decrement in psychomotor development in Spain i.e. a country where prevention of malaria is not needed [39]. Ventilation has been shown to be important for reduction of the indoor concentrations of semi-volatile organic compounds such as formaldehyde [40]. A prospective study in Sweden reported low ventilation rates in the home as one of the significant risk factors for autism spectrum disorders [41].

Some recommendations aim at exposure during outdoor activities. Herbicides were observed to be associated with an increase in Non-Hodgkin lymphoma and glyphosate, the most used herbicide, was classified by the International Agency for Research on Cancer as probably carcinogenic to humans [42, 43]. Long-term exposure to air pollution PM2.5 was associated with non-accidental cardiovascular mortality, lung cancer and chronic obstructive pulmonary disease in a recent Chinese study, with risks being higher than estimated from previous cohort studies in western Europe and North America [44]. Exercising outdoors may increase exposure to urban air pollution [45]. Intense traffic in a highway tunnel causes an important exposure and significant biological effects [46]. Interestingly, the number of particles downwind of traffic decreases exponentially with the distance and is substantially lower at a distance of 200 meter [47].

Ionizing radiation is an important carcinogen, and prenatal exposure to diagnostic x-rays is associated with increased prevalence of childhood cancer [48, 49].

**References**

1. Filippini G, Farinotti M, and Ferrarini M. Active and passive smoking during pregnancy and risk of central nervous system tumours in children. Paediatr.Perinat.Epidemiol. 2000;14:78-84.
2. Julvez J, Ribas-Fito N, Torrent M, Forns M, Garcia-Esteban R, Sunyer J. Maternal smoking habits and cognitive development of children at age 4 years in a population-based birth cohort. Int.J.Epidemiol. 2007;36:825-832.
3. Irner TB. Substance exposure in utero and developmental consequences in adolescence: a systematic review. Child Neuropsychol. 2012;18:521-549.
4. Polanska K, Jurewicz J, and Hanke W. Smoking and alcohol drinking during pregnancy as the risk factors for poor child neurodevelopment - A review of epidemiological studies. Int.J.Occup.Med.Environ.Health 2015;28:419-443.
5. Evlampidou I, Bagkeris M, Vardavas C, Koutra K, Patelarou E, Koutis A. et al. Prenatal Second-Hand Smoke Exposure Measured with Urine Cotinine May Reduce Gross Motor Development at 18 Months of Age. J.Pediatr. 2015;167:246-252.
6. Tabrizi MM and Bidgoli SA. Increased risk of childhood acute lymphoblastic leukemia (ALL) by prenatal and postnatal exposure to high voltage power lines: a case control study in Isfahan, Iran. Asian Pac.J.Cancer Prev. 2015;16:2347-2350.
7. Schuz J. Exposure to extremely low-frequency magnetic fields and the risk of childhood cancer: update of the epidemiological evidence. Prog Biophys Mol Biol, 2011;107: 339-342.
8. Birks L, Guxens M, Papadopoulou E, Alexander J, Ballester F, Estarlich M, et al. Maternal cell phone use during pregnancy and child behavioral problems in five birth cohorts. Environ.Int. 2017;104:122-131.
9. Zarei S, Mortazavi SM, Mehdizadeh AR, Jalalipour M, Borzou S, Taeb S et al. A Challenging Issue in the Etiology of Speech Problems: The Effect of Maternal Exposure to Electromagnetic Fields on Speech Problems in the Offspring. J.Biomed.Phys.Eng 2015;5:151-154.
10. Szczepańska N, Namieśnik J, Kudłak B. [Assessment of toxic and endocrine potential of substances migrating from selected toys and baby products.](https://vpn.gw.ulg.ac.be/pubmed/,DanaInfo=www.ncbi.nlm.nih.gov,SSL+27662857)Environ Sci Pollut Res Int. 2016;23:24890-24900.
11. Zhang Y, Yu C, Wang L. [Temperature exposure during pregnancy and birth outcomes: An updated systematic review of epidemiological evidence.](https://vpn.gw.ulg.ac.be/pubmed/,DanaInfo=www.ncbi.nlm.nih.gov,SSL+28284544) Environ Pollut. 2017;225:700-712.
12. Den Hond E, Paulussen M, Geens T, Bruckers L, Baeyens W, David F et al. Biomarkers of human exposure to personal care products: Results from the Flemish Environment and Health Study (FLEHS 2007–2011). Sci. Total Environ. 463–2013;464:102–110.
13. Frederiksen H, Nielsen JKS, Mørck TA, Hansen PW, Jensen JF, Nielsen O et al. Urinary excretion of phthalate metabolites, phenols and parabens in rural and urban Danish mother–child pairs. Int. J. Hyg. Environ. Health 2013;216:772–783.
14. Patel S. Fragrance compounds: The wolves in sheep’s clothings. Med. Hypotheses 2017;102:106–111.
15. Marie C, Lémery D, Vendittelli F, Sauvant-Rochat MP. [Perception of Environmental Risks and Health Promotion Attitudes of French Perinatal Health Professionals.](https://vpn.gw.ulg.ac.be/pubmed/,DanaInfo=www.ncbi.nlm.nih.gov,SSL+27999342) Int J Environ Res Public Health. 2016;13:383-398.
16. Engel E, Santarelli F, Vasold R, Maisch T, Ulrich H, Prantl L et al. [Modern tattoos cause high concentrations of hazardous pigments in skin.](https://vpn.gw.ulg.ac.be/pubmed/,DanaInfo=www.ncbi.nlm.nih.gov,SSL+18353031) Contact Dermatitis 2008;58:228-233.
17. Philippat C, Nakiwala D, Calafat AM, Botton J, De Agostini M, Heude B et al. EDEN Mother–Child Study Group. [Prenatal Exposure to Nonpersistent Endocrine Disruptors and Behavior in Boys at 3 and 5 Years.](https://vpn.gw.ulg.ac.be/pubmed/,DanaInfo=www.ncbi.nlm.nih.gov,SSL+28937960) Environ Health Perspect. 2017;125:097014. doi: 10.1289/EHP1314
18. Vernet C, Pin I, Giorgis-Allemand L, Philippat C, Benmerad M, Quentin J, et al. EDEN Mother–Child Cohort Study Group. [In Utero Exposure to Select Phenols and phthalates and Respiratory Health in Five-Year-Old Boys: A Prospective Study.](https://vpn.gw.ulg.ac.be/pubmed/,DanaInfo=www.ncbi.nlm.nih.gov,SSL+28934727) Environ Health Perspect. 2017;125:097006. doi: 10.1289/EHP1015
19. Bonde JP, Flachs EM, Rimborg S, Glazer CH, Giwercman A, Ramlau-Hansen CH et al. [The epidemiologic evidence linking prenatal and postnatal exposure to endocrine disrupting chemicals with male reproductive disorders: a systematic review and meta-analysis.](https://vpn.gw.ulg.ac.be/pubmed/,DanaInfo=www.ncbi.nlm.nih.gov,SSL+27655588) Hum Reprod Update. 2016;23:104-125.
20. Philips EM, Jaddoe VW, Trasande L. [Effects of early exposure to phthalates and bisphenols on cardiometabolic outcomes in pregnancy and childhood.](https://vpn.gw.ulg.ac.be/pubmed/,DanaInfo=www.ncbi.nlm.nih.gov,SSL+27596818) Reprod Toxicol. 2017;68:105-118.
21. Brender JD, Weyer PJ. [Agricultural Compounds in Water and Birth Defects.](https://vpn.gw.ulg.ac.be/pubmed/,DanaInfo=www.ncbi.nlm.nih.gov,SSL+27007730) Curr Environ Health Rep. 2016;3:144-152.
22. Kyriklaki A, Vafeiadi M, Kampouri M, Koutra K, Roumeliotaki T, Chalkiadaki G et al. [Prenatal exposure to persistent **organic** pollutants in association with offspring neuropsychological development at 4years of age: The Rhea mother-child cohort, Crete, Greece.](https://vpn.gw.ulg.ac.be/pubmed/,DanaInfo=www.ncbi.nlm.nih.gov,SSL+27666324) Environ Int. 2016;97:204-211.
23. Simões-Wüst AP, Moltó-Puigmartí C, Jansen EH, van Dongen MC, Dagnelie PC, Thijs C. [Organic food consumption during pregnancy and its association with health-related characteristics: the KOALA Birth Cohort Study.](https://vpn.gw.ulg.ac.be/pubmed/,DanaInfo=www.ncbi.nlm.nih.gov,SSL+28625206) Public Health Nutr. 2017;20:2145-2156.
24. Brantsæter AL, Torjusen H, Meltzer HM, Papadopoulou E, Hoppin JA, Alexander J et al. [Organic Food Consumption during Pregnancy and Hypospadias and Cryptorchidism at Birth: The Norwegian Mother and Child Cohort Study (MoBa).](https://vpn.gw.ulg.ac.be/pubmed/,DanaInfo=www.ncbi.nlm.nih.gov,SSL+26307850) Environ Health Perspect. 2016;124:357-364.
25. Bouvard V, Loomis D, Guyton KZ, Grosse Y, Ghissassi FE, Benbrahim-Tallaa L et al. International Agency for Research on Cancer Monograph Working Group. [Carcinogenicity of consumption of red and processed meat.](https://vpn.gw.ulg.ac.be/pubmed/,DanaInfo=www.ncbi.nlm.nih.gov,SSL+26514947) Lancet Oncol. 2015;16:1599-1600
26. Fu Z, Deming SL, Fair AM, Shrubsole MJ, Wujcik DM, Shu XO et al. Well-done meat intake and meat-derived mutagen exposures in relation to breast cancer risk: the Nashville Breast Health Study. Breast Cancer Res.Treat. 2011;129:919-928.
27. Kleinjans J, Botsivali M, Kogevinas M, Merlo DF; NewGeneris consortium. [Fetal exposure to dietary carcinogens and risk of childhood cancer: what the NewGeneris project tells us.](https://www.ncbi.nlm.nih.gov/pubmed/26320143) BMJ 2015;351:h4501
28. Swedish Chemicals Agency. Hazardous chemical substances in textiles – proposals for risk management measures. 2016. <https://www.kemi.se/en/directly-to/publications/reports>.
29. Avagyan R, Luongo G, Thorsén G, Östman C. Benzothiazole, benzotriazole, and their derivates in clothing textiles - a potential source of environmental pollutants and human exposure. Environ Sci Pollut Res Int. 2015;22:5842-5849.
30. Limpiteeprakan P, Babel S, Lohwacharin J, Takizawa S. Release of silver nanoparticles from fabrics during the course of sequential washing. Environ Sci Pollut Res Int. 2016;23:22810-22818.
31. Lynge E, Anttila A, Hemminki K. Organic solvents and cancer. Cancer Causes Control 1997;8:406-419.
32. McKinney PA, Raji OY, van Tongeren M, Feltbower RG. The UK Childhood Cancer Study: maternal occupational exposures and childhood leukaemia and lymphoma. Radiat. Prot. Dosimetry. 2008;132:232-240.
33. Le Cornet C, Fervers B, Pukkala E, Tynes T, Feychting M, Hansen J et al. Parental Occupational Exposure to Organic Solvents andesticular Germ Cell Tumors in their Offspring: NORD-TEST Study. Environ Health Perspect. 2017;125:067023. doi: 10.1289/EHP864. PubMed PMID: 28893722.
34. Franck U, Weller A, Röder SW, Herberth G, Junge KM, Kohajda T et al. Prenatal VOC exposure and redecoration are related to wheezing in early infancy. Environ Int. 2014;73:393-401.
35. Kim S, Hong SH, Bong CK, Cho MH. Characterization of air freshener emission: the potential health effects. J Toxicol Sci. 2015;40:535-50.
36. Trantallidi M, Dimitroulopoulou C, Wolkoff P, Kephalopoulos S, Carrer P. EPHECT III: Health risk assessment of exposure to household consumer products. Sci Total Environ. 2015;536: 903-913.
37. Mitro SD, Dodson RE, Singla V, Adamkiewicz G, Elmi AF, Tilly MK et al. Consumer Product Chemicals in Indoor Dust: A Quantitative Meta-analysis of U.S.Studies. Environ Sci Technol. 2016;50:10661-10672.
38. Roberts JW, Wallace LA, Camann DE, Dickey P, Gilbert SG, Lewis RG et al. Monitoring and reducing exposure of infants to pollutants in house dust. Rev Environ Contam Toxicol. 2009;201:1–39.
39. [Llop S](https://vpn.gw.ulg.ac.be/pubmed/,DanaInfo=www.ncbi.nlm.nih.gov,SSL+?term=Llop%20S%5BAuthor%5D&cauthor=true&cauthor_uid=23831543), [Julvez J](https://vpn.gw.ulg.ac.be/pubmed/,DanaInfo=www.ncbi.nlm.nih.gov,SSL+?term=Julvez%20J%5BAuthor%5D&cauthor=true&cauthor_uid=23831543), [Fernandez-Somoano A](https://vpn.gw.ulg.ac.be/pubmed/,DanaInfo=www.ncbi.nlm.nih.gov,SSL+?term=Fernandez-Somoano%20A%5BAuthor%5D&cauthor=true&cauthor_uid=23831543), [Santa Marina L](https://vpn.gw.ulg.ac.be/pubmed/,DanaInfo=www.ncbi.nlm.nih.gov,SSL+?term=Santa%20Marina%20L%5BAuthor%5D&cauthor=true&cauthor_uid=23831543), [Vizcaino E](https://vpn.gw.ulg.ac.be/pubmed/,DanaInfo=www.ncbi.nlm.nih.gov,SSL+?term=Vizcaino%20E%5BAuthor%5D&cauthor=true&cauthor_uid=23831543), [Iñiguez C](https://vpn.gw.ulg.ac.be/pubmed/,DanaInfo=www.ncbi.nlm.nih.gov,SSL+?term=I%C3%B1iguez%20C%5BAuthor%5D&cauthor=true&cauthor_uid=23831543) et al. Prenatal and postnatal insecticide use and infant neuropsychological development in a multicenter birth cohort study. [Environ Int.](https://vpn.gw.ulg.ac.be/pubmed/,DanaInfo=www.ncbi.nlm.nih.gov,SSL+23831543) 2013;59:175-182.
40. Liu C, Zhang Y, Benning JL, Little JC. The effect of ventilation on indoor exposure to semivolatile organic compounds. Indoor Air. 2015;25:285-296.
41. Larsson, M., Weiss, B., Janson, S., Sundell, J., Bornehag, C.-G. Associations between indoor environmental factors and parental-reported autistic spectrum disorders in children 6–8 years of age. Neurotoxicology 2009;30:822–831.
42. IARC Working Group Glyphosate. Some organophosphate insecticides and herbicides: diazinon, glyphosate, malathion, parathion, and tetrachlorvinphos. IARC Monogr Prog, 2015;112:1–92.
43. Myers JP, Antoniou MN, Blumberg B, Carroll L, Colborn T, Everett LG et al. Concerns over use of glyphosate-based herbicides and risks associated with exposures: a consensus statement. Environ.Health 2016;15:1944.
44. Giles LV and Koehle MS. 2014. The health effects of exercising in air pollution. Sports Med. 44:223-249.
45. Yin P, Brauer M, Cohen A, Burnett RT, Liu J, Liu Y et al. Long-term Fine Particulate Matter Exposure and Nonaccidental and Cause-specific Mortality in a Large National Cohort of Chinese Men. Environ.Health Perspect. 2017;125:117002. doi: 10.1289/EHP1673.
46. Bos I, De Boever P, Emmerechts J, Buekers J, Vanoirbeek J, Meeusen R, et al. Changed gene expression in brains of mice exposed to traffic in a highway tunnel. Inhal.Toxicol*.* 2012;24:676-686.
47. Zhu,Y, Kuhn T, Mayo P, and Hinds WC. Comparison of daytime and nighttime concentration profiles and size distributions of ultrafine particles near a major highway. Environ.Sci.Technol. 2006;40:2531-2536.
48. Doll R, Wakeford R. Risk of childhood cancer from fetal irradiation. Br J Radiol 1997;70:130-139.
49. Wakeford R, Little MP. Risk coefficients for childhood cancer after intrauterine irradiation: a review. Int. J. Radiat. Biol. 2003;79: 293-309.

**Table 1B. Some recommendations aiming at reduced exposure to health hazards during pregnancy and early postnatal life**

|  | **Recommendations** | **Targeted hazards** | | | **References** |
| --- | --- | --- | --- | --- | --- |
|  |  | **EDCs** | **Mutagens** | **Others** |  |
| Every-  where | Stop smoking tobacco and drinking alcohol | x | x | x | [2-4] |
|  | Limit as much as possible passive smoking | x | x |  | [1, 5] |
|  | Avoid frequent close presence to power lines; limit the use of cell phones or cordless mobile phones |  |  | x | [6-9] |
|  | Limit the use of plastic or rubber toys and prefer products declared to be free of bisphenol A or phthalates | x |  |  | [10] |
|  | Stay in a cool place in case of heat > 30°C |  |  | x | [11] |
| Personal care | Restrict the use of cosmetics and lotions as much as possible | x |  |  | [12, 13] |
|  | Prioritize unscented products and stop using perfumes | x |  |  | [14] |
|  | Do not color your hair; do not polish your nails | x |  |  | [15] |
|  | Avoid tattoos |  | x |  | [16] |
| Food and drinks | Prioritize food and drinks from glass container instead of plastic bottles or metal cans | x |  |  | [17-20] |
|  | Do not microwave food in plastic recipients | x |  |  |  |
|  | Use quality-controlled water in glass bottles | x |  | x | [21] |
|  | Prioritize organic food whenever possible | x | x |  | [22-24] |
|  | Avoid processed, especially nitrite treated, meat |  | x |  | [25] |
|  | Avoid charred meat and consumption of bread or other cereal products that are darkened due to high temperature treatment |  | x |  | [26, 27] |
| Home care | Wash new clothes before wearing them | x | x | x | [28-30] |
|  | Avoid exposure to organic solvents |  | x |  | [31-33] |
|  | Avoid as much as possible painting or coating (walls, doors, floors, …) | x | x |  | [34] |
|  | Avoid scented cleansing products , air fresheners and fragrances | x | x | x | [35, 36] |
|  | Clean inside the house using damp clothes and reduce dust | x |  |  | [37, 38] |
|  | Do not use insecticides | x |  |  | [39] |
|  | Ventilate the bedrooms and living rooms at home for 10 min, 1-2 times a day | x |  | x | [40, 41] |
| Outdoor | Avoid the use of herbicides or pesticides | x | x |  | [42, 43] |
|  | Close the car windows and recycle air while driving on highways, in tunnels and in heavy traffic |  | x | x | [44, 46, 47] |
|  | Prefer exercising in green areas and avoid heavily polluted air such as within 200 meters of heavy traffic |  | x | x | [44, 45] |
| Others | Avoid exposure to medical x-rays unless really necessary |  | x | x | [48, 49] |
